# Recent Trends in Surgical Strategies of Early-Stage Gallbladder Cancer: A Narrative Review

**DOI:** 10.3390/jcm14155483

**Published:** 2025-08-04

**Authors:** Junseo Choi, Ji Su Kim, Jun Suh Lee

**Affiliations:** 1 School of Medicine, Konkuk University, Seoul 05029, Republic of Korea; xvzc@med.kku.ac.kr; 2 Department of Surgery, Incheon St. Mary’s Hospital, College of Medicine, The Catholic University of Korea, Seoul 21431, Republic of Korea; 3 Department of Surgery, Bucheon Sejong Hospital, Bucheon 14754, Republic of Korea

**Keywords:** gallbladder neoplasms, cholecystectomy, lymph node excision, hepatectomy, minimally invasive surgical procedures

## Abstract

**Background/Objectives:** Gallbladder cancer (GBC) is a lethal malignancy curable only by surgical resection in early stages (Tis, T1, T2). Significant controversy exists regarding the optimal extent of surgery. This review summarizes recent trends and evidence on surgical strategies for Tis, T1, and T2 GBC to guide practice and research. **Methods:** This narrative review synthesizes recent literature on surgical management of Tis, T1a, T1b, and T2 GBC based on American Joint Committee on Cancer (AJCC) 8th edition staging. It examines simple vs. extended cholecystectomy (simple cholecystectomy (SC) vs. extended/radical cholecystectomy (EC/RC)), the role of lymphadenectomy (LND) and hepatectomy, and minimally invasive surgery (MIS). **Results:** Simple cholecystectomy is curative for Tis/T1a GBC. For T1b, regional LND is essential for staging/potential benefit, especially examining ≥5–6 nodes. Tumor size is critical; SC alone may suffice for T1b < 1 cm (low lymph node metastasis (LNM) risk), while EC/RC with LND is indicated for ≥1 cm (higher LNM risk). Routine hepatectomy for T1b lacks survival support. For T2 GBC, mandatory regional LND (≥6 nodes) is required for both T2a and T2b substages due to high LNM rates; T2b has higher LNM than T2a. Routine hepatectomy for T2 is debated; evidence suggests no routine benefit for T2a beyond LND, with conflicting findings for T2b. R0 resection is paramount. MIS is feasible for early stages in experienced hands. **Conclusions:** Management of early GBC is moving towards risk stratification. SC is standard for Tis/T1a. Adequate regional LND is crucial for T1b (especially ≥1 cm) and mandatory for T2 GBC. Routine hepatectomy, particularly for T2b, remains controversial. Tailored surgery prioritizes R0 resection and comprehensive LND, necessitating further standardized research.

## 1. Introduction

Gallbladder cancer (GBC) is the most common malignancy of the biliary tract, accounting for the majority of cases in this system [1]. It is highly lethal, often diagnosed at an advanced stage due to vague symptoms and silent progression [2]. As a result, long-term survival remains poor, with limited improvements over recent decades [2]. However, with the widespread adoption of laparoscopic cholecystectomy for benign disease, early-stage GBC is increasingly detected incidentally.

This shift in presentation has highlighted the need for evidence-based surgical strategies tailored to T-stage, tumor biology, and perioperative findings. Surgical resection is currently the only curative treatment, particularly when GBC is detected at an early, localized stage [3]. Achieving a complete (R0) resection is essential for improving survival outcomes. However, the ideal surgical approach—especially the extent of liver and lymph node resection—remains controversial, even in early-stage disease [3].

This review aims to synthesize recent literature on surgical strategies for early-stage GBC, with particular attention to tumor stage, tumor size, lymphovascular invasion, and the distinction between incidental and preoperatively diagnosed cases. We also discuss the evolving role of minimally invasive techniques and propose risk-stratified approaches to optimize outcomes and inform future research.

## 2. Background: Staging of GBC

Accurate staging is essential in GBC, guiding surgical planning and predicting prognosis [4]. Due to its aggressive biology and early spread, determining the extent of disease is critical for selecting candidates for curative resection [5].

The AJCC tumor-node-metastasis (TNM) system classifies GBC based on tumor invasion depth (T), nodal status (N), and distant metastasis (M) [4]. This review focuses on early-stage disease, defined as Tis, T1a, T1b, and T2 by the AJCC 8th edition [6].

**Figure 1:**
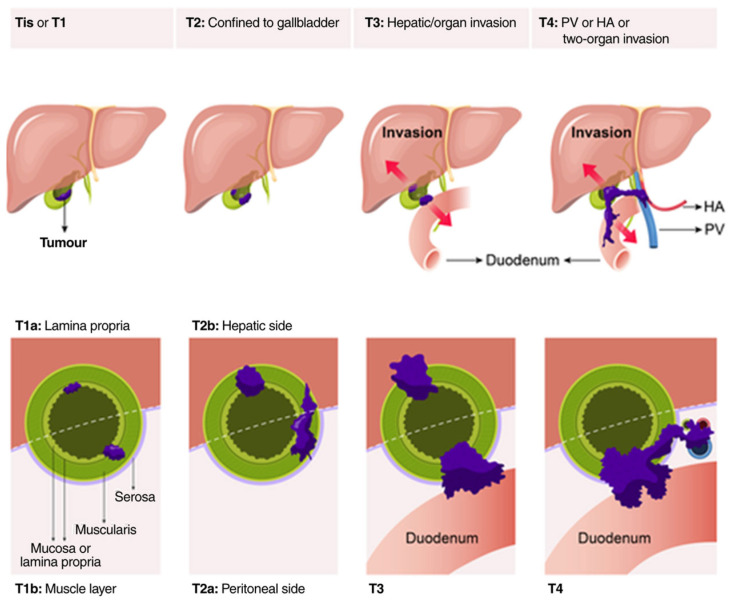
AJCC (8th Edition) TNM staging of gallbladder cancer [3].

Tis indicates carcinoma in situ, limited to the epithelial layer without lamina propria invasion [6]. T1 tumors extend into the lamina propria (T1a) or muscular layer (T1b), without reaching the perimuscular connective tissue [5]. T2 tumors invade the perimuscular connective tissue but do not breach the serosa or involve the liver [6]. Importantly, T2 is subclassified into T2a (peritoneal side without involvement of the serosa) and T2b (hepatic side with no extension into the liver), reflecting prognostic differences [7]. T staging of GBC is shown in Figure 1.

These depth-based distinctions directly influence the surgical approach—from simple cholecystectomy in Tis/T1a to radical resection with lymphadenectomy for T1b and T2 disease [5].

In patients with suspected gallbladder cancer, contrast-enhanced CT or MRI is recommended to evaluate the extent of disease and exclude obvious liver or lymph node involvement. CA 19–9 and CEA may provide supplementary information, although their sensitivity is limited in early-stage disease. Intraoperatively, surgeons may consider laparoscopic total biopsy or frozen section analysis when malignancy is suspected, though frozen section has limited accuracy in distinguishing between T1a and T1b lesions. Tokumitsu et al. reported on laparoscopic total biopsy as a staged strategy, allowing for definitive pathology prior to deciding on extended surgery [8]. Open EC and LND should be reserved for confirmed or strongly suspected cases in high-risk patients or where intraoperative findings are concerning.

## 3. Surgical Management of Tis and T1a GBC

For Tis (carcinoma in situ) and T1a GBC, SC is considered both definitive and curative [9,10,11]. In Tis, malignant cells are confined to the epithelial layer without invasion of the lamina propria. T1a lesions extend into the lamina propria but not beyond [4,6]. These early stages carry an extremely low risk of lymphatic or vascular spread, and recurrence after complete gallbladder removal is rare [9].

### Simple Cholecystectomy

SC involves removal of the gallbladder, including ligation of the cystic duct and artery at Calot’s triangle, and separation from the cystic plate [4]. In Tis and T1a disease, this approach achieves excellent outcomes, with a 5-year survival rate (5-YSR) near 100% [6,9].

The key to oncologic success is meticulous pathological review of the specimen. Full-thickness sampling and margin assessment are critical to confirm the stage and ensure complete resection [6]. Particular attention should be paid to the cystic duct margin. If tumor cells are present, reoperation to excise the extrahepatic bile duct may be necessary to prevent residual disease and local recurrence [12].

However, routine bile duct resection (BDR) in the absence of margin involvement is controversial. Some studies report no clear survival benefit and caution against added surgical morbidity [4].

The use of robotic cholecystectomy in suspected or early-stage GBC remains controversial. While the robotic platform offers enhanced dexterity and visualization—particularly beneficial for lymphadenectomy and hepatic resections—there is currently insufficient evidence supporting its superiority over conventional laparoscopy or open surgery for oncologic outcomes in GBC. Its use should be limited to centers with expertise in robotic hepatobiliary surgery and carefully selected patients.

## 4. Surgical Management of T1b GBC

### 4.1. Surgical Approaches for T1b GBC: Extended/Radical Cholecystectomy

EC typically involves a wedge resection of the gallbladder fossa or a formal segment IVb/V liver resection, depending on tumor location and surgeon preference. Lymphadenectomy generally includes stations 12 (pericholedochal), 8 (common hepatic artery), and 13 (retropancreatic), which represent the primary nodal drainage pathways of the gallbladder. Adequate lymph node retrieval (≥6 nodes) is considered essential for accurate staging and prognosis.

- **Hepatectomy (Hep)**: Resection of adjacent liver, from wedge resection (e.g., 2 cm margin) to anatomic segments IVb/V [5]. Major hepatectomy is usually for more advanced disease [13].
- **Lymphadenectomy (LND)**: Dissection of regional nodes, typically hepatoduodenal ligament (Station 12), common hepatic artery (Station 8), and retro-pancreatic/retro-duodenal (Station 13) [13]. Some extend to para-aortic nodes (Station 16) [13]. The national comprehensive cancer network (NCCN) includes porta hepatis, gastrohepatic ligament, and retroduodenal nodes.
- **BDR**: Not routine for T1b; reserved for positive cystic duct margin or direct tumor involvement [13]. Benefit is uncertain [14].
- **Port Site Resection**: Generally not recommended for incidental GBC after laparoscopy; does not improve survival [5]. Incidence of port-site metastasis is ~10% [15].

Inconsistent terminology (EC, RC, extended surgical resection (ESR), radical resection) hinders comparison [13], thus this article will use EC/RC broadly for SC + LND +/− hepatectomy.

### 4.2. The Extent of Cholecystectomy: SC vs. EC/RC

Evidence comparing SC and EC/RC for T1b GBC is conflicting.

#### 4.2.1. Survival Outcomes

- **Evidence Suggesting No Significant Survival Benefit for EC/RC**: Several reviews and database analyses find no clear survival advantage for EC/RC. Lee et al.’s review found “no definite evidence” favoring EC [15]. Kim et al.’s meta-analysis showed comparable cancer-related death rates (eecurrence rate (RR): 1.06; 95% confidence interval (CI): 0.93–1.22; *p* = 0.36) [16]. Surveillance, epidemiology, and end results (SEER) analyses often report similar overall survival/cancer-specific survival (OS/CSS); one found median OS 48 mo (SC) vs. 38 mo (EC; *p* = 0.791) [17]. Cohort studies also report comparable overall survival/disease-specific survival (OS/DSS). [18] A recent National Cancer Database (NCDB) analysis found median OS 89.5 mo (SC) vs. 91.4 mo (RC; *p* = 0.55) and no increased mortality hazard for SC (hazardous ratio (HR): 1.23; *p* = 0.12) [19].
- **Evidence Suggesting Potential Survival Benefit for EC/RC**: Other studies suggest that EC/RC improves survival. Lee et al.’s review included studies showing better 5-YSR for EC (e.g., 79% vs. 42%, *p* = 0.03; 100% vs. 37.5%, *p* < 0.01) [15]. A Cochrane review indicated better survival with radical resection (Hep + LND) vs. SC [14]. A decision analysis projected a 3.43-year survival benefit for RC [20]. Some SEER analyses report advantages: one found longer median OS with RC (101.7 mo) vs. SC + LND (87.6 mo) vs. SC (71.3 mo; *p* < 0.05); [18] another noted better 5-YSR for T1 overall with RC vs. SC (79% vs. 50%; *p* < 0.01) [18]. Individual studies also report better survival with extended resection [18].

Conflict likely stems from methodological limitations (selection bias, confounding, inconsistent definitions, heterogeneity) [17]. Any benefit might be small, subgroup-specific, or obscured by these issues [21]. All provided evidence is tabulated below, in Table 1.

#### 4.2.2. Recurrence Rates and Patterns

EC/RC may reduce recurrence. Pooled analysis showed lower recurrence after EC (2.7%) vs. SC (12.5%; *p* < 0.01) [15]. A propensity-matched study found all recurrences (11.1%) in the SC group, half being nodal [22]. Another study noted similar overall rates but more loco-regional recurrence after SC [23]. Higher recurrence (especially nodal) after SC supports arguments for LND [22]. EC/RC might provide better local/regional control even without consistent OS benefit.

#### 4.2.3. Preoperative Morbidity and Mortality

EC/RC involves longer operative times and hospital stays [22]. Pooled data suggested higher complication rates (28.0% EC (N = 75) vs. 21.2% SC (N = 52)) and slightly higher mortality (1.5% vs. 1.0%) [15]. LND morbidity alone is generally considered low [24]. Laparoscopic EC/RC is feasible, may shorten stays, and shows comparable outcomes in experienced hands [25]. Some studies initially reported higher laparoscopic complication rates [26], but many now show equivalence [27].

**Table 1:** Comparison of outcomes between SC and EC/RC in T1b GBC [Table A1].

Study	Definition of EC/RC	Number of Patients		Outcome(s)	Result (SC vs. EC/RC)		Statistical Significance (*p*-Value)
		SC	EC/RC		SC	EC/RC	
Rhodin et al., 2024 [19]	RC	187	763	Median OS	89.5 mo.	91.4 mo.	*p* = 0.55
Xu et al., 2020 [17]	SC + Hep	218	183	Median OS	48 mo.	38 mo.	*p* = 0.791
				Median CSS	48 mo.	36 mo.	*p* = 0.736
Liu et al., 2018 [18]	RC (SC + LND + Hep); C + L (SC + LND)	562	98 (RC), 231 (C + L)	Median OS	71.3 mo.	101.7 mo. (RC) 87.6 mo. (C + L)	*p* < 0.05
Yuza et al., 2020 [18]	RC (SC + LND ± Hep)	29	18	10-yr OS	66%	64%	*p* = 0.618
				10-yr DSS	100%	86%	*p* = 0.151
Goetz et al., 2014 [18]	RR	56	28	5-YSR	34%	75%	*p* = 0.01
Yoon et al., 2014 [22]	EC	36	18	5-YSR	88.8%	93.3%	*p* = 0.521
Hari et al., 2013 [18]	RC	1115		5-YSR (T1 overall)	50%	79%	*p* < 0.01
Lee et al., 2011 [15]	SC + LND + Hep (>wedge) ± other organs	375	185	Pooled Recurrence Rate	12.5%	2.7%	*p* < 0.01
Rhodin et al., 2024 [19]	RC	187	763	Mortality Hazard	HR 1.23 (SC vs. RC)		*p* = 0.12

### 4.3. The Role and Extent of Hepatectomy

The necessity of routine hepatectomy (wedge or segments IVb/V) in T1b GBC is increasingly questioned [5]. A Chinese cohort study (77 T1b patients with SC + LND) found no significant 5-year OS improvement with added hepatectomy (79.5% with hep vs. 76.1% without; *p* = 0.50) [28]. This suggests that hepatectomy may not add survival benefit if adequate LND is done. The large multicenter, retrospective operative management of GBC (OMEGA) cohort study on 3767 patients also found that liver resection did not improve long-term survival (overall survival/recurrence-free survival (OS/RFS)) for any T-stage (including T1b) and increased morbidity/mortality [29]. Even in node-negative (N0) patients, no benefit was seen [29]. Despite this, some guidelines still recommend hepatectomy (wedge or IVb/V) for T1b [5]. The rationale is ensuring R0 margins, especially for hepatic-side tumors or positive cystic duct margins [13]. Some surgeons use a selective approach [13]. Routine significant liver resection for all T1b GBC seems hard to justify based on survival alone. Its role may be limited to ensuring R0 status, weighed against increased morbidity [29].

Hepatectomy usually involves either non-anatomical wedge resection or anatomical resection of segments IVb and V. While both achieve negative margins, the survival benefit of more extensive resection in T1b remains unclear. Some studies suggest that in carefully selected patients, SC with lymphadenectomy may be adequate if nodal involvement is absent [12,14]. Tumor location also influences decision-making—lesions near the cystic plate may justify liver resection, whereas peritoneal-side lesions might not [9,12].

### 4.4. The Role and Extent of Lymphadenectomy

#### 4.4.1. Necessity for Staging and Potential Therapeutic Benefit

Accurate N staging is crucial for prognosis and adjuvant therapy decisions [13]. Most guidelines recommend LND for T1b+ [5]. Omitting LND risks under-staging [30]. LND may also offer therapeutic benefit by removing micro-metastases [22].

#### 4.4.2. Impact of LND on Survival Outcomes

Studies support a survival benefit for LND in T1b GBC. SEER analyses show improved OS/CSS with LND [17]. One reported median OS 69 mo. (LND) vs. 37 mo. (no LND; *p* = 0.051, trend) [17]. SC + LND shows significant advantage over SC alone [17]. A Chinese study found higher 5-year OS with SC + LND (76.3%) vs. SC alone (56.8%; *p* = 0.036); SC + LND was an independent predictor of improved OS (HR 0.51, 95% CI 0.26–0.99) [28]. Even node-positive patients can achieve long-term survival (5-year DSS 43% reported) with R0 resection and aggressive LND [31]. The impact of LND in T1b GBC is abbreviated in Table 2.

#### 4.4.3. Optimal Extent: Number of Nodes Examined and Prognostic Significance

SEER data showed that T1b SC patients with five or more nodes excised had significantly better OS (adjusted hazardous ratio (aHR) 0.231, *p* = 0.004) and CSS (aHR 0.183, *p* = 0.018) vs. no LND; one to four nodes showed no significant advantage [17], highlighting the importance of adequate LND. Retrieving six or more nodes is often recommended [30]. Even when no cancer has spread to the lymph nodes (N0 patients), the number of lymph nodes removed and examined can still provide important information about a patient’s likely outcome [30], since examining more nodes can sometimes reveal small amounts of cancer that were initially missed, leading to a more accurate staging of the disease. Additionally, the lymph node ratio (LNR), which is the number of positive nodes divided by the total number of nodes examined, is emerging as a useful prognostic factor [13].

#### 4.4.4. Lymphatic Drainage Pathways and Rational Extent

Understanding lymphatic drainage guides LND extent [31]. GBC has high propensity for lymphatic spread [31]. Primary drainage is along the cystic/common bile duct (Station 12), then potentially retropancreatic (Station 13) and para-aortic (Station 16), also ascending along the hepatic artery (Station 8) [13]. The first echelon nodes are the cystic duct/pericholedochal; the second echelon nodes include the hepatic artery, portal vein, and retropancreatic [31]. Rational LND should cover first/second echelons (Stations 12, 8, 13), aligning with NCCN. Some advocate extending to para-aortic (Station 16) [1], but the majority of literature only requires Stations 12, 8, and 13 for LND. Adequate LND (≥5–6 nodes) appears critical for staging and therapeutic benefit in T1b GBC.

**Table 2:** Impact of LND (extent/number of nodes) on outcomes in T1b GBC [Table A2].

Study	Comparison Groups	Outcome(s)	Result
Choi et al., 2013 [32]	No. of lymph nodes (LNs) examined	Prognosis	Total LNs examined implicated
Fong et al., 2017 [14]	No LND vs. LND	Survival	LND benefit suggested
Fan et al., 2018 [33]	No. of LNs examined	OS	More nodes examined = better OS
Xu et al., 2020 [17]	No LND vs. LND	Median OS	37 mo vs. 69 mo (*p* = 0.051, trend)
		Median CSS	35 mo vs. 46 mo (*p* = 0.281)
	SC–LND vs. SC + LND (≥5 nodes)	OS	LND ≥ 5 nodes better (aHR 0.231, *p* = 0.004)
		CSS	LND ≥ 5 nodes better (aHR 0.183, *p* = 0.018)
	SC–LND vs. SC + LND	OS	SC + LND better (*p* = 0.024)
Jin et al., 2021 [28]	SC vs. SC + LND	5-yr OS	56.8% vs. 76.3% (*p* = 0.036)
		OS (Multivariate)	SC + LND better (HR 0.51, *p* = 0.036, 95% CI: 0.26–0.99)
Mayo et al., 2022 [34]	LNs examined (1–2 vs. ≥6)	Therapeutic Index (LNM% × 3 yr OS)	6.9 vs. 16.9

### 4.5. Factors Including Surgical Strategy and Outcomes

#### 4.5.1. Tumor Size

Tumor size is critical for risk stratification [18]. T1b tumors < 1 cm have very low/negligible LNM rates (0% in several SEER analyses) [35]. For T1b < 1 cm, studies show no significant survival benefit (OS/CSS) for EC/LND vs. SC alone [35]. T1b tumors ≥ 1 cm have significantly higher LNM rates (e.g., 14.4% reported) [35]. For T1b ≥ 1 cm, EC/LND is associated with improved OS vs. SC alone [35]. This suggests that SC alone may be adequate for T1b < 1 cm, while EC/RC including LND is justified for T1b ≥ 1 cm [18].

#### 4.5.2. Lymphovascular Invasion (LVI)

LVI is an adverse prognostic factor, associated with recurrence (*p* = 0.028) and cancer-related death in one T1 GBC study [23]. T1b patients with LVI had worse 5-year disease-free survival (DFS) (45.7% vs. 83.6%; *p* = 0.046) [23]. However, the same study found no significant survival benefit for EC vs. SC in the T1b + LVI subgroup (5-year DFS, *p* = 0.054; 5-year OS, *p* = 0.091), possibly due to small sample size [23]. LVI is reported as uncommon [15] or absent [11] in some T1b cohorts. While indicating aggressive biology, LVI’s role in choosing SC vs. EC/RC needs more study; it likely reinforces the need for LND if present.

#### 4.5.3. Incidental Versus Preoperatively Diagnosed GBC

Many T1b cases are incidental gallbladder cancers (iGBC), which are a type of gallbladder cancer that is discovered unexpectedly during or, more commonly, after a cholecystectomy [5]. For T1b + iGBC, re-resection (EC/RC) is generally recommended if fit and no distant metastases [13]. Laparoscopic re-resection is feasible with comparable outcomes [27], but one study suggested worse OS with continued laparoscopy versus conversion to open for intraoperatively detected iGBC (T1b or higher) [36]. Thus, management trends towards selective re-resection should be evaluated based on risk [37].

#### 4.5.4. Other Factors

Patient age and gender [11], histological grade [5], and potentially CA 19–9 levels [34] can influence prognosis. Tumor location (hepatic vs. peritoneal) is key in T2 [18] but less established for T1b [23]. Tumor size (<1 cm vs. ≥1 cm) appears to be the most robust factor currently for guiding LND necessity in T1b GBC [18].

### 4.6. Conclusion: Surgical Strategies for T1b GBC

Management of T1b GBC is complex. Evidence conflicts on EC/RC vs. SC survival benefit. LND is critical for staging and likely therapeutic, especially with five to six or more nodes examined. Routine hepatectomy lacks strong support and adds morbidity [29]. Tumor size < 1 cm suggests low LNM risk, potentially allowing SC alone, while ≥1 cm warrants LND [18].

A risk-stratified approach:

- **T1a GBC**: SC standard.
- **T1b GBC < 1 cm (R0, no LVI)**: SC alone may be reasonable.
- **T1b GBC ≥ 1 cm or Positive/Uncertain Margins or LVI**: Extended resection including adequate LND (six or more nodes) strongly recommended. Hepatectomy as needed for R0 margins.
- **Incidental T1b GBC discovered after cholecystectomy**: Re-resection (EC/RC with LND ± tailored hepatectomy) is generally recommended if the tumor is ≥1 cm and/or LVI is present. For tumors < 1 cm without LVI and with clear margins, observation may be considered, though decisions should be individualized. This reflects similar principles applied in preoperatively diagnosed cases.

Laparoscopic EC/RC is feasible [25]. High-quality prospective studies (RCTs or comparative effectiveness) are needed, stratified by tumor size/LVI. Standardized definitions and reporting are crucial. Further validation of the <1 cm criterion and evaluation of laparoscopic vs. open outcomes are needed.

Management is shifting towards risk-based paradigms, potentially de-escalating surgery for low-risk tumors and optimizing extended resections by focusing on adequate LND over routine hepatectomy. Careful selection, multidisciplinary discussion, and critical appraisal of data are key.

## 5. Surgical Management of T2 GBC

The 8th edition AJCC staging manual significantly advanced GBC understanding by subclassifying T2 tumors (invading peri-muscular connective tissue without breaching serosa or invading liver) into T2a (peritoneal side) and T2b (hepatic side) [38]. This was based on studies showing worse outcomes for T2b tumors, potentially due to the lack of a serosal barrier on the hepatic side facilitating earlier spread [18].

### 5.1. T2a vs. T2b: The Effect of Tumor Location

The T2a/T2b subclassification reflects observed prognostic differences linked to tumor location [18]. The hepatic side’s anatomical vulnerability—lacking a serosal barrier and potentially exhibiting denser subserosal lymphovascular structures—may contribute to the higher rate of nodal metastasis seen in T2b. This suggests that both anatomical proximity to the liver and inherent tumor biology likely interact to create a more aggressive clinical profile in T2b cases [39].

#### 5.1.1. Comparative Clinicopathology

Significant clinicopathological differences exist between T2a and T2b GBC:

- **Lymph Node (LN) Metastasis**: T2b GBC consistently shows significantly higher LN metastasis rates than T2a GBC across studies [40]. Examples include 37.9% (T2b) vs. 29.5% (T2a) [40], 48.0% vs. 17.1% [41], 46% vs. 20% [41], and 36.6% vs. 26.6% [40]. Overall LN involvement in T2 GBC is high, up to 46% [3] or even 45–80% for T2–T4 stages [41].
- **Other Pathological Features**: T2b tumors are also associated with higher rates of vascular and perineural invasion (though not always statistically significant) [39], potentially larger size, and poorer differentiation [39].

#### 5.1.2. Survival Outcomes

These differences often impact survival, though tumor location’s independent prognostic role is debated:

- **Evidence Supporting Worse T2b Prognosis**: Many studies report significantly poorer OS, DSS, and RFS for T2b vs. T2a GBC [39]. Examples: 5-year OS 80.7% (T2b) vs. 96.8% (T2a) [3]; 5-year DSS 65.4% vs. 74.8% [40]; 3-year RFS 54.5% vs. 67.7% [4]. Meta-analyses show significantly higher mortality HRs for T2b vs. T2a (e.g., HR 2.141 [40], HR 3.16 [42], HR 13.62 for OS [39]).
- **Evidence Questioning Prognostic Independence**: Some analyses find that the T2a/T2b survival difference diminishes or loses significance after adjusting for factors like LN status [41]. One large study found that T2b location was not an independent prognostic factor in multivariate analysis; only LN metastasis was (HR 3.222) [43]. Another identified LN metastasis, vascular invasion, and tumor location as independent factors [3]. Others found no significant survival difference [18] or noted that differences disappeared when stratified by nodal status [40].

#### 5.1.3. Controversies and Caveats

Inconsistencies may arise from varying T2a/T2b definitions [39], different surgical procedures [39], confounding from adjuvant therapy [41], exclusion of Nx patients [40], and limitations of retrospective studies [39].

Despite controversies, the consistently higher LN metastasis rate in T2b tumors marks them as biologically more aggressive [40]. While T2b location might not always be independently prognostic after accounting for metastasis [43], it signals increased risk. The attenuation of survival differences when stratified by nodal status highlights LN involvement’s paramount importance [38]. Accurate nodal staging via adequate LND is critical for all T2 GBC, potentially outweighing the T2a/T2b distinction once the N-stage is known [40].

### 5.2. The Role and Extent of Lymphadenectomy

Regional LND is standard for T1b/T2 GBC, crucial for staging and potential therapy [6]. Omitting LND (Nx status) carries a poor prognosis similar to N1 disease [4].

#### 5.2.1. LND and Survival Outcomes

Studies support LND’s value in T2 GBC:

- Overall Survival Benefit: LND is associated with improved survival in T2 GBC compared to no LND [38]. Similar benefits seen in T1b GBC, especially with five or more nodes removed [38].
- Benefit in Node-Negative (N0) Patients: SEER data showed that LND’s survival benefit in T2 GBC was particularly evident in N0 patients, possibly due to removing micro-metastases or better staging [38].
- Uncertain Benefit in Node-Positive (N1) Patients: The same SEER analysis found no significant survival benefit from LND in N1 patients, perhaps because N1 status itself dictates prognosis, or benefits are obscured [38].

Impact of LND in T2 GBC is abbreviated in Table 3.

#### 5.2.2. Extent of LND

Optimal LND extent is debated:

- Number of Nodes Retrieved: More extensive LND (higher node count) may improve outcomes and staging accuracy. Thresholds like four or more [38] or five or more nodes [38] are linked to better survival. AJCC recommends examining six or more nodes for adequate N staging [44]. Lymph node ratio (LNR) > 0.28 is associated with worse OS [41].
- Definition of Regional vs. Extended LND: Regional LND includes nodes along the cystic duct, common bile duct, hepatic artery, and portal vein (N1) [38]. Extended LND might include peri-pancreatic, celiac, SMA, or para-aortic nodes (N2) [38]. Terminology varies.

#### 5.2.3. Differential Impact in T2a vs. T2b

Data mainly address LND necessity in T2 GBC overall, not comparing different LND extents specifically for T2a vs. T2b. The key difference is T2b’s higher LN metastasis incidence [40], making adequate LND crucial for staging T2b. No strong evidence suggests that a fundamentally different LND extent is needed for T2b vs. T2a based solely on T-substage for accurate N-staging. Achieving adequate regional dissection (six or more nodes) seems paramount for both [44].

LND’s critical prognostic role [38], association with improved survival [38], and poor prognosis with Nx status [4] mandate its inclusion in T2 GBC surgery. Given high occult nodal metastasis rates (especially T2b) [40] and N-stage’s impact [43], adequate regional LND (six or more nodes) appears non-negotiable for potentially curative T2 GBC management, regardless of T-substage or hepatectomy decisions [44].

**Table 3:** LND outcomes in T2 GBC.

Study	Patient Cohort	Comparison	Key Outcome(s)	Key Finding Summary
Zhang et al., 2021 [38]	T2	Regional LND (RL) vs. No RL	OS	Significant survival benefit with RL overall.
	T2 (stratified by N stage)	RL vs. No RL	OS	Benefit observed in N0 stage, but not in N1 stage.
	T2 (RL patients)	≥4 nodes vs. 1–3 nodes	OS	Significantly better OS with ≥4 nodes removed.
Chen et al. (referenced in [41])	Advanced GBC (six or more nodes retrieved)	LNR > 0.28 vs. ≤0.28	OS	LNR > 0.28 associated with worse median OS (18 vs. 27.5 months, *p* = 0.004).

### 5.3. The Role and Extent of Hepatectomy

Hepatic resection’s role in T2 GBC surgery is highly debated, with conflicting evidence [6]. Guidelines often advocate for EC including liver resection [18], but studies challenge its necessity and benefit, especially its routine use across T2 substages [38]. For this section, the term hepatectomy is used to describe both non-anatomical wedge resection (wedge resection, 2–3 cm margin) or anatomical segmentectomy (segmentectomy, commonly IVb + V) [39].

#### 5.3.1. Hepatectomy Outcomes in T2a GBC

Evidence strongly suggests no routine benefit for hepatectomy in T2a GBC. Substantial literature indicates that EC offers no significant survival advantage over SC + LND for T2a GBC [6]. Multiple studies report no significant difference in OS, DSS, RFS, or DFS [6]. Example: 5-year DSS 81.8% (EC) vs. 73.7% (SC + LND), *p* = 0.361 [43]. Several reports conclude that hepatic resection is unnecessary for T2a GBC; LND without hepatectomy is sufficient [39].

#### 5.3.2. Hepatectomy Outcomes in T2b GBC

The situation for T2b GBC is complex due to conflicting findings:

- **Evidence Supporting Benefit**: Several studies suggest potential survival advantage with hepatic resection for T2b GBC [3]. Some report significant survival improvement (e.g., *p* = 0.029 [3]; 5-yr OS 80.3% with liver resection vs. 30.0% without, *p* = 0.032 [45]; better OS with EC vs. SC/SC + LND [18]), others show positive trends (e.g., 5-yr DSS 71.7% vs. 59.3% [43]). A meta-analysis found liver resection linked to higher 5-year OS odds in T2b (OR 2.20) [42]. One study found that segment IVb + V resection yielded better 3-year survival than wedge resection for T2b (72.7% vs. 41.6%) [3].
- **Evidence Against Benefit**: Other analyses (database/multicenter studies) found no significant survival benefit from adding hepatic resection, even for T2b [6]. These concluded that survival was not superior with hepatectomy vs. LND alone [39], DFS was similar, [6] EC did not improve prognosis [38], and EC/SC + LND had comparable outcomes [40]. Multivariable analyses in large cohorts failed to identify liver resection as an independent prognostic factor [6].

Hepatectomy outcomes in T2 GBC is abbreviated in Table 4.

#### 5.3.3. Wedge Resection vs. Segmentectomy

Optimal hepatectomy type is debated, with limited data. Some analyses suggest comparable survival between wedge and segment IVb/V resection for T2 GBC overall [45]. One matched study (EC with bi-segmentectomy (ECB) vs. EC with wedge resection (ECW) for T2/T3) found no RFS/OS difference, but ECB had less blood loss and fewer complications [40]. Conversely, one T2b-specific study reported superior 3-year survival with segment IVb + V resection vs. wedge resection [3].

#### 5.3.4. Complications and Morbidity

Surgical extent impacts perioperative outcomes. Major hepatectomy or adjacent organ resection increases major complication/death risk, often without improving long-term survival [4]. EC with liver resection had greater blood loss and longer stays than LND alone in one analysis [6]. Optimal postoperative course (textbook outcomes in liver surgery, TOLS) was achieved in only ~50% of GBC patients undergoing curative resection and was linked to less extensive surgery (wedge vs. major hepatectomy, T1 vs. higher T, N0 vs. N+) [29]. ECW had higher complication rates than ECB [40].

Evidence suggests diminishing returns for hepatic resection in T2 GBC. For T2a, multiple studies conclude that adding hepatectomy to adequate LND does not consistently improve survival [6]. Given added risks/morbidity [6], routine hepatectomy for T2a seems hard to justify. Prognosis appears tied to clear margins and negative nodes via cholecystectomy + LND.

For T2b, ambiguity persists [6]. Conflicting results question if benefits seen in some studies are real or confounded. The strong T2b-LN metastasis link [44] makes disentangling effects difficult. Lack of consistent benefit suggests that routine hepatectomy may not be needed for all T2b patients either [6].

**Table 4:** Hepatectomy outcomes in T2a and T2b GBC.

Study	Patient Cohort	Comparison	Key Outcome(s)	Key Finding Summary
Park et al. (2020) [43]	T2a	EC vs. SC + LND	5-yr DSS	No significant difference (81.8% vs. 73.7%, *p* = 0.361).
	T2b			Trend towards better survival with EC (71.7% vs. 59.3%, *p* = 0.057).
Lee et al. (2020) [3]	T2a	Hepatectomy vs. No Hepatectomy	Survival Rate	No difference (*p* = 0.320).
	T2b			Better survival with hepatectomy (*p* = 0.029).
Choi et al. (2019) [39]	T2a and T2b	LND + Hepatectomy vs. LND without Hepatectomy	Survival (OS/DFS)	No significant difference in survival regardless of tumor location (T2a or T2b). Hepatectomy not superior.
OGBY-GBC Collaborative (2023) [4]	T2 (overall)	Liver Resection vs. Cholecystectomy Alone	RFS, OS (multivariable)	No significant improvement associated with liver resection (wedge, segmentectomy, or major hepatectomy).
	T2 (overall)	Wedge/Segmentectomy vs. Cholecystectomy Alone	RFS (univariable subgroup)	Improved RFS associated with wedge (HR 0.59) and segmentectomy (HR 0.78) compared to cholecystectomy alone (*p* < 0.0001). Note: Contradicts multivariable analysis.
Zhang et al. (SEER data, 2021) [38]	T2 (overall)	EC vs. SC (Propensity Score matching (PSM))	Median OS	No significant difference (17 vs. 15 months, *p* = 0.258 after matching). Extended cholecystectomy did not significantly improve prognosis.
Kim et al. (Meta-analysis, 2021) [40]	T2a	EC vs. SC + LND	Survival	No significant difference (OR 0.802).
	T2b			No significant difference (OR 0.820).
Khan et al. (Meta-analysis, 2021) [42]	T2a	Liver Resection vs. No Liver Resection	5-yr OS	No additional survival benefit.
	T2b			Significantly higher odds of 5-yr OS with liver resection (OR 2.20).
Kim et al. (2022) [18]	T2a	EC vs. SC vs. SC + LND	OS	No difference among surgery methods.
	T2b			EC showed better OS than SC (*p* = 0.043) and SC + LND (*p* = 0.003).
Lee et al. (2017) [45]	T2b	LND + Liver Resection vs. LND without Liver Resection	5-yr OS	Significantly greater survival with liver resection (80.3% vs. 30.0%, *p* = 0.032). Extent (wedge/segmentectomy) did not matter (*p* = 0.526). LND without liver resection was poor prognostic factor.
Zhang et al. (2023) [3]	T2b	Segment IVb + V Resection vs. Wedge Resection	3-yr Survival	Higher survival with segmentectomy (72.7% vs. 41.6%).
Jain et al. (2021) [40]	T2 and T3	Bi-segmentectomy (ECB) vs. Wedge (ECW)	RFS, OS, Complications	No significant difference in RFS/OS (*p* = 0.264/*p* = 0.161). ECB had less blood loss (*p* = 0.005) and fewer complications (*p* = 0.035).
Kim et al. (2023) [6]	T2 (PSM)	LND + L vs. LND alone	5-yr DFS	No significant difference overall (*p* = 0.376) or in T2a (*p* = 0.988)/T2b (*p* = 0.196) subgroups. LND + L more blood loss, longer stay.

### 5.4. Surgical Strategy for T2 GBC

Integrating findings shows a complex picture for T2 GBC management. Evidence strongly supports adequate regional LND (six or more nodes) for staging and potential therapeutic benefit across all T2 GBC [38]. Nx status correlates with significantly worse outcomes [4].

Routine hepatic resection’s benefit remains highly contentious. For T2a GBC, considerable evidence suggests hepatectomy adds little survival benefit beyond cholecystectomy + adequate LND [6]. Unlike T1b tumors, where tumor size has been associated with nodal metastasis and recurrence, the role of tumor size in T2a GBC remains poorly defined in the literature. As such, current recommendations for T2a do not include size-based stratification for hepatectomy. Further studies evaluating tumor size as a prognostic factor in T2a are needed to guide more nuanced surgical decision-making. SC + LND could be standard for confirmed T2a, reserving hepatectomy for margin clearance. Lack of consistent benefit plus risks [6] suggests that routine hepatectomy may overtreat many T2a patients.

For T2b GBC, the optimal strategy is unclear due to conflicting data. Some studies show improved survival with hepatectomy [6,18,38,39,40,46]; others find no advantage over LND alone. [6] This suggests that T2b is not monolithic regarding surgical needs. Factors beyond location (invasion depth, proximity to structures, microscopic liver involvement, nodal patterns) might influence hepatectomy’s benefit. Current T2b classification may lack granularity.

Other powerful prognostic factors in T2 GBC include LN metastasis (most critical, independent predictor) [6], achieving R0 resection [6], vascular invasion [3], perineural invasion [6], poor differentiation [3], larger tumor size [38], elevated tumor markers (CA19–9, CEA, CA125) [44], and older age [6]. Adjuvant chemotherapy shows potential benefit, especially for node-positive patients [43].

- Accurate Staging: Use rigorous imaging and intraoperative assessment to differentiate T2a/T2b, acknowledging limitations [39].
- Mandatory Lymphadenectomy: Perform regional LND (goal of six or more nodes examined [44]) standardly for curative-intent T2a/T2b surgery [38].
- Surgical Strategy for T2a GBC: Consider cholecystectomy + regional LND without routine hepatectomy as appropriate for most pathologically confirmed T2a GBC, reserving hepatectomy for R0 margin needs [6].
- Surgical Strategy for T2b GBC: Individualize hepatectomy decision due to conflicting evidence [3,4,6,18,29,38,39,40,41,43,44,46,47,48,49]. Both cholecystectomy + LND alone [6] and EC including hepatectomy + LND [6,18,38,39,40,46,47,48] are supported. Consider perceived liver involvement risk, R0 likelihood, comorbidities, and expertise.
- Prioritize R0 Resection: Primary goal is complete tumor removal with negative margins [6].

## 6. Emerging Trends in GBC Surgery: Minimally Invasive Surgery

Minimally invasive surgery (MIS), including laparoscopic and robotic techniques, is increasingly applied to early-stage gallbladder cancer (GBC), particularly Tis, T1a, and select T1b cases [4,5]. MIS offers advantages such as reduced blood loss, shorter hospital stays, and faster recovery compared to open surgery [4,5].

For Tis and T1a, where simple cholecystectomy is often curative, laparoscopic approaches achieve comparable oncologic outcomes to open procedures [4,48]. However, historical concerns persist regarding bile spillage and port-site recurrence in cases where malignancy is suspected preoperatively or discovered incidentally [4]. Current best practices recommend the use of specimen retrieval bags to mitigate these risks [5].

In T1b disease, where regional lymphadenectomy is needed, MIS—including robotic platforms—has shown promise in achieving R0 resection and adequate lymph node harvest in high-volume centers [2,4,5]. Still, oncologic safety remains paramount, and surgeon experience is critical. Proper case selection is essential to balance minimal invasiveness with complete cancer clearance [4].

## 7. Conclusions

Gallbladder cancer remains a highly lethal malignancy, with surgical resection offering the only potential for cure in early stages. For Tis (carcinoma in situ) and T1a lesions, simple cholecystectomy is widely accepted as definitive and curative due to the low risk of lymphatic spread. Managing T1b GBC presents more complexity; while evidence comparing simple and extended/radical cholecystectomy (EC/RC) regarding overall survival is conflicting, regional lymphadenectomy is deemed essential for accurate staging and likely provides therapeutic benefit, especially when five to six nodes or more are examined. Tumor size significantly influences risk in T1b, with tumors ≥ 1 cm having a higher risk of lymph node metastasis (LNM) and potentially benefiting from EC/RC with LND, whereas SC alone may be adequate for tumors < 1 cm. Routine hepatectomy for T1b lacks strong survival support and increases morbidity. For T2 GBC, the distinction between T2a (peritoneal side) and T2b (hepatic side) is clinically significant due to T2b’s higher LNM rates, necessitating mandatory regional lymphadenectomy (six or more nodes) for both substages. However, the necessity of routine hepatectomy for T2 GBC remains debated, with evidence suggesting little consistent survival benefit for T2a beyond LND, while findings for T2b are contradictory. Minimally invasive surgery is increasingly explored for early stages, demonstrating feasibility in experienced hands. Overall, management is shifting towards risk stratification, prioritizing adequate LND and R0 resection, while tailoring the extent of hepatectomy, necessitating further high-quality, standardized prospective research.

## Data Availability

Not applicable.

## Abbreviations

The following abbreviations are used in this manuscript:

GBC	gallbladder cancer
MIS	minimally invasive surgery
AJCC	American Joint Committee on Cancer
TNM	tumor-node-metastasis
SC	simple cholecystectomy
EC	extended cholecystectomy
RC	radical cholecystectomy
ESR	extended surgical resection
Ch	cholecystectomy
LND	lymphadenectomy
NCCN	National Comprehensive Cancer Network
BDR	bile duct resection
OS	overall survival
CSS	cancer-specific survival
DFS	disease-free survival
5-YSR	5-year survival rate
DSS	disease-specific survival
RR	relative risk
CI	confidence interval
SEER	Surveillance, Epidemiology, and End Results
NCDB	National Cancer Database
HR	hazard ratio
Hep	hepatectomy
NS	not significant
PSM	propensity score matching
RL	regional lymphadenectomy
RFS	recurrence-free survival
LNM	lymph node metastasis
LNs	lymph nodes
aHR	adjusted hazard ratio
LVI	lymphovascular invasion
OR	odds ratio
ECB	extended cholecystectomy with bi-segmentectomy
ECW	extended cholecystectomy with wedge resection
TOLS	textbook outcomes in liver surgery

## Appendix A. Data Collected for Surgical Treatment of T1b GBC

**Table A1:** Comparison of outcomes between SC and EC/RC in T1b GBC.

Study (Author, Year)	Study Type	Definition of EC/RC Used	No. of T1b Patients (SC/EC)	Primary Outcome(s)	Result (SC vs. EC/RC)	Statistical Significance (*p*-Value)
Lee et al., 2011 [15]	Systematic Review (29 Retro studies)	SC + LND + Liver (>wedge) +/− other organs	375/185	Pooled Recurrence Rate	12.5% vs. 2.7%	*p* < 0.01
	Systematic Review (subset)	Varied	Varied	5-yr OS	e.g., 42% vs. 79%; 37.5% vs. 100%	*p* = 0.03; *p* < 0.01 (in specific studies)
Kim et al., 2017 [16]	Meta-analysis (22 articles)	Varied (SC vs. EC)	Total 2578 (all T1)	Cancer-related Death (T1b)	RR 1.06 (EC higher risk, NS)	*p* = 0.36
Xu et al., 2020 [17]	SEER (2004–13)	ESR (SC + Liver)	218/183 (ESR)	Median OS	48 mo vs. 38 mo	*p* = 0.791
				Median CSS	48 mo vs. 36 mo	*p* = 0.736
Yuza et al., 2020 [18]	Cohort (Japan)	RC (SC + LND +/− Hep)	29/18	10-yr OS	66% vs. 64%	*p* = 0.618
				10-yr DSS	100% vs. 86%	*p* = 0.151
Yoon et al., 2014 [22]	Cohort (Korea, PSM)	EC (SC + LND)	36/18	5-YSR	88.8% vs. 93.3%	*p* = 0.521
				Recurrence Rate	11.1% vs. 0%	N/A (All in SC)
Jensen et al., 2020 [50]	Cohort (Chile)	EC	86/43	5-yr OS	Comparable (83% overall)	NS
Rhodin et al., 2024 [19]	NCDB (2004–18)	RC	187/763	Median OS	89.5 mo vs. 91.4 mo	*p* = 0.55
				Mortality Hazard	HR 1.23 (SC vs. RC)	*p* = 0.12
Fong et al., 2017 [14]	Cochrane Review	Radical Resection (Hep + LND)	Varied	Survival	Significantly better vs. SC	*p*-value not stated
Abramson et al., 2009 [20]	Decision Analysis	RC	Simulated	Life Expectancy Gain	+3.43 years vs. SC	N/A (Model)
Liu et al., 2018 [18]	SEER	RC (SC + LND + Hep); C + L (SC + LND)	562/231/98	Median OS	71.3 vs. 87.6 vs. 101.7 mo	*p* < 0.05 (overall)
Hari et al., 2013 [18]	SEER	RC	Varied	5-yr Survival (T1 overall)	50% vs. 79%	*p* < 0.01
Goetz et al., 2014 [18]	Retrospective	Radical Resection	56/28	5-yr Survival (T1b)	34% vs. 75%	*p* = 0.01
Zhang et al. [18]	Retrospective	EC	Varied	Survival	Better vs. SC (87.5% vs. 61.3%)	*p*-value not stated

**Table A2:** Impact of LND (extent/number of nodes) in outcomes in T1b GBC.

Study	Study Type	Comparison Groups	Outcome(s)	Result	Statistical Significance (*p*-Value)
Xu et al., 2020 [17]	SEER (2004–13)	No LND vs. LND	Median OS	37 mo vs. 69 mo	*p* = 0.051 (trend)
			Median CSS	35 mo vs. 46 mo	*p* = 0.281
		SC no LND vs. SC + LND (≥5 nodes)	OS	LND ≥ 5 nodes better	aHR 0.231 (*p* = 0.004)
			CSS	LND ≥ 5 nodes better	aHR 0.183 (*p* = 0.018)
		SC no LND vs. SC + LND	OS	SC + LND better	*p* = 0.024
Jin et al., 2021 [28]	Multicenter Cohort (China)	SC vs. SC + LND	5-yr OS	56.8% vs. 76.3%	*p* = 0.036
			OS (Multivariate)	SC + LND better	HR 0.51 (*p* = 0.036, 95% CI 0.26–0.99)
Fong et al., 2017 [14]	Cochrane Review	No LND vs. LND	Survival	LND benefit suggested	*p*-value not stated
Mayo et al., 2022 [34]	Multi-institutional Cohort (US)	LNs examined (1–2 vs. ≥6)	Therapeutic Index (LNM% x 3yr OS)	6.9 vs. 16.9	N/A (Index comparison)
Fan et al. [33]	SEER (N0 patients)	No. of LNs examined	OS	More nodes examined = better OS	*p*-value not stated
Kim et al. [32]	Cohort (Korea, N0 patients)	No. of LNs examined	Prognosis	Total LNs examined implicated	*p*-value not stated

**Table A3:** Summary of LNM rates and association with tumor size/LVI in T1b GBC.

Study	Study Type	No. of T1b Patients	Overall LNM Rate (%)	LNM Rate by Tumor Size (<1 cm/≥1 cm)	LNM Rate by LVI Status (+/−)	Key Finding/Conclusion
Lee et al., 2011 [15]	Systematic Review	560	10.9%	Not Specified (NS)	NS (LVI rare)	LNM risk exists in T1b, unlike T1a (1.8%).
Xu et al., 2020 [17]	SEER (2004–13)	277 (127 had LND)	14.8% (overall cohort)/11.8% (15/127 LND pts)	0% (0/23)/14.4% (15/104)	NS	LNM risk significant only for tumors ≥ 1 cm. RL may be necessary for tumors > 1 cm.
Wang et al., 2019 [51]	SEER (2004–15)	277 (127 had LND)	11.8% (15/127 LND pts)	0% (0/23)/14.4% (15/104)	NS	SC adequate for T1b < 1 cm; EC beneficial for T1b ≥ 1 cm due to LNM risk.
Jin et al., 2021 [28]	Multicenter Cohort (China)	121 (77 had LND)	9.1% (7/77 LND pts)	NS	NS	LND associated with better OS regardless of LNM status found.
Yuza et al., 2020 [18]	Cohort (Japan)	47 (18 had LND)	0% (0/18 LND pts)	NS	1 patient had LVI	No LNM found in this cohort; SC outcomes similar to RC.
You et al., 2008 [18]	Cohort (Korea)	25	8.0% (2/25)	NS	1 patient had LVI	LNM occurred in T1b; recommended SC + LND for T1b.
Butte et al., 2011 [24]	Review	Literature	Up to 20%	NS	NS	LNM risk justifies mandatory LND for T1b.
Vo et al., 2019 [35]	NCDB (2004–12)	464 (217 had EC + RL)	~15% (in EC + RL group)	0%/NS (>1 cm)	NS	LNM risk justifies EC + RL; no LNM if <1 cm.
Park et al., 2019 [23]	Cohort (Korea)	22	0% (0/8 had LND)	NS	36.4% (8/22) had LVI	No LNM found; LVI associated with worse DFS (*p* = 0.048).
Liu et al., 2018 [18]	SEER	891	10.9% (implied from Lee ref)	NS	NS	Supports RC for T1b based on OS difference vs. SC.
